# Supplementary material for: Clopidogrel responder status is uninfluenced by CYP2C19*2 in Danish patients with stroke
Source: PLoS One. 2020 Dec 28;15(12):e0236260. doi: 10.1371/journal.pone.0236260 (PMC7769274; doi:10.1371/journal.pone.0236260)
Supplement: S1 Data — (DOCX) [file pone.0236260.s001.docx]

Data table 1.

| Patient ID-number | Date of inclusion | Age | Gender | Diabetes mellitus | Hypertension | PRU | Inhibition | Statin treatment | PPI |
| --- | --- | --- | --- | --- | --- | --- | --- | --- | --- |
| 1 | 2016-05-24 | 1959-07 | 0 | 0 | 0 | 9 | 96 | 1 | 0 |
| 2 | 2016-05-25 | 1948-09 | 0 | 0 | 1 | 0 | 100 | 1 | 0 |
| 3 | 2016-05-25 | 1960-02 | 0 | 0 | 0 | 76 | 71 | 1 | 1 |
| 4 | 2016-05-26 | 1954-06 | 1 | 0 | 1 | 53 | 70 | 1 | 0 |
| 5 | 2016-05-27 | 1955-09 | 0 | 0 | 1 | 52 | 75 | 1 | 1 |
| 6 | 2016-05-27 | 1939-05 | 0 | 0 | 1 | 129 | 48 | 1 | 0 |
| 7 | 2016-06-03 | 1961-09 | 1 | 0 | 0 | 67 | 77 | 0 | 0 |
| 8 | 2016-06-07 | 1966-05 | 0 | 0 | 1 | 143 | 42 | 1 | 0 |
| 9 | 2016-06-07 | 1938-10 | 0 | 0 | 1 | 7 | 97 | 0 | 1 |
| 10 | 2016-08-15 | 1967-10 | 0 | 0 | 0 | 4 | 98 | 1 | 0 |
| 11 | 2016-08-15 | 1945-03 | 0 | 1 | 1 | 6 | 97 | 1 | 1 |
| 12 | 2016-08-18 | 1938-07 | 0 | 0 | 0 | 5 | 97 | 1 | 1 |
| 13 | 2016-08-31 | 1959-10 | 0 | 0 | 1 | 100 | 54 | 1 | 1 |
| 14 | 2016-09-07 | 1941-09 | 0 | 0 | 0 | 163 | 27 | 1 | 0 |
| 15 | 2016-09-09 | 1946-04 | 0 | 0 | 1 | 87 | 58 | 1 | 0 |
| 16 | 2016-09-09 | 1973-11 | 0 | 0 | 0 | 195 | 15 | 1 | 0 |
| 17 | 2016-09-19 | 1956-06 | 0 | 0 | 0 | 6 | 97 | 1 | 0 |
| 18 | 2016-09-21 | 1960-07 | 0 | 1 | 0 | 33 | 82 | 1 | 0 |
| 19 | 2016-09-21 | 1968-10 | 0 | 0 | 0 | 46 | 78 | 1 | 0 |
| 20 | 2016-09-28 | 1951-05 | 1 | 0 | 0 | 3 | 98 | 1 | 1 |
| 21 | 2016-10-03 | 1943-03 | 1 | 0 | 0 | 66 | 67 | 1 | 0 |
| 22 | 2016-10-14 | 1956-03 | 0 | 1 | 1 | 137 | 36 | 1 | 0 |
| 23 | 2016-10-14 | 1935-02 | 0 | 0 | 1 | 9 | 96 | 1 | 0 |
| 24 | 2016-10-14 | 1935-03 | 1 | 0 | 1 | 181 | 22 | 0 | 1 |
| 25 | 2016-10-14 | 1949-02 | 1 | 0 | 0 | 98 | 56 | 1 | 0 |
| 26 | 2016-10-26 | 1967-09 | 1 | 0 | 0 | 135 | 21 | 1 | 0 |
| 27 | 2016-10-28 | 1964-04 | 0 | 0 | 0 | 162 | 4 | 1 | 0 |
| 28 | 2016-11-02 | 1958-10 | 1 | 1 | 0 | 64 | 69 | 1 | 1 |
| 29 | 2017-01-20 | 1948-05 | 0 | 0 | 1 | 110 | 51 | 1 | 0 |
| 30 | 2017-01-23 | 1945-10 | 1 | 0 | 0 | 161 | 35 | 0 | 0 |
| 31 | 2017-01-26 | 1952-09 | 0 | 0 | 0 | 42 | 79 | 1 | 1 |
| 32 | 2017-01-26 | 1925-11 | 0 | 0 | 0 | 68 | 74 | 1 | 0 |
| 33 | 2017-01-26 | 1949-03 | 1 | 0 | 0 | 161 | 27 | 1 | 1 |
| 34 | 2017-01-27 | 1947-03 | 1 | 0 | 1 | 118 | 42 | 1 | 0 |
| 35 | 2017-01-27 | 1929-04 | 1 | 0 | 0 | 6 | 97 | 1 | 0 |
| 36 | 2017-01-27 | 1947-07 | 1 | 0 | 1 | 135 | 32 | 1 | 0 |
| 37 | 2017-02-01 | 1937-08 | 1 | 1 | 1 | 140 | 38 | 1 | 1 |
| 38 | 2017-02-01 | 1946-11 | 0 | 0 | 1 | 129 | 55 | 1 | 1 |
| 39 | 2017-02-02 | 1954-06 | 1 | 0 | 1 | 63 | 71 | 1 | 0 |
| 40 | 2017-02-02 | 1931-04 | 1 | 0 | 1 | 145 | 39 | 1 | 1 |
| 41 | 2017-02-02 | 1943-06 | 1 | 0 | 1 | 120 | 47 | 1 | 1 |
| 42 | 2017-02-03 | 1945-09 | 0 | 0 | 0 | 126 | 39 | 0 | 0 |
| 43 | 2017-02-03 | 1934-05 | 0 | 1 | 1 | 202 | 5 | 0 | 1 |
| 44 | 2017-02-03 | 1940-05 | 1 | 1 | 0 | 110 | 59 | 1 | 0 |
| 45 | 2017-02-03 | 1970-12 | 0 | 0 | 0 | 99 | 56 | 0 | 0 |
| 46 | 2017-02-06 | 1935-03 | 0 | 0 | 0 | 117 | 43 | 1 | 1 |
| 47 | 2017-02-06 | 1943-08 | 0 | 0 | 1 | 49 | 69 | 1 | 1 |
| 48 | 2017-02-07 | 1941-09 | 0 | 0 | 1 | 141 | 7 | 1 | 0 |
| 49 | 2017-02-09 | 1947-06 | 1 | 0 | 0 | 7 | 97 | 1 | 1 |
| 50 | 2017-02-09 | 1931-05 | 1 | 0 | 0 | 163 | 18 | 1 | 1 |
| 51 | 2017-02-20 | 1945-03 | 0 | 0 | 0 | 54 | 59 | 1 | 0 |
| 52 | 2017-02-20 | 1947-12 | 0 | 0 | 0 | 29 | 86 | 1 | 0 |
| 53 | 2017-03-01 | 1956-12 | 1 | 0 | 0 | 123 | 44 | 0 | 1 |
| 54 | 2017-03-02 | 1968-07 | 0 | 0 | 0 | 37 | 77 | 1 | 0 |
| 55 | 2017-03-02 | 1944-03 | 1 | 0 | 0 | 52 | 71 | 1 | 1 |
| 56 | 2017-03-03 | 1956-03 | 0 | 0 | 1 | 201 | 8 | 1 | 0 |
| 57 | 2017-03-03 | 1957-11 | 0 | 1 | 1 | 107 | 35 | 0 | 0 |
| 58 | 2017-03-03 | 1944-04 | 0 | 0 | 1 | 111 | 45 | 1 | 0 |
| 59 | 2017-03-21 | 1942-03 | 1 | 0 | 0 | 90 | 60 | 1 | 0 |
| 60 | 2017-03-21 | 1955-12 | 1 | 0 | 0 | 120 | 56 | 1 | 0 |
| 61 | 2017-04-20 | 1959-01 | 0 | 0 | 0 | 62 | 69 | 1 | 1 |
| 62 | 2017-04-25 | 1950-12 | 0 | 0 | 0 | 82 | 55 | 1 | 1 |
| 63 | 2017-04-25 | 1947-11 | 0 | 0 | 0 | 98 | 48 | 1 | 0 |
| 64 | 2017-04-26 | 1937-08 | 0 | 0 | 0 | 103 | 41 | 1 | 0 |
| 65 | 2017-04-26 | 1943-04 | 0 | 0 | 1 | 46 | 74 | 1 | 0 |
| 66 | 2017-04-27 | 1968-08 | 0 | 0 | 0 | 9 | 94 | 1 | 0 |
| 67 | 2017-04-27 | 1945-04 | 0 | 0 | 1 | 9 | 98 | 0 | 0 |
| 68 | 2017-04-27 | 1954-09 | 1 | 0 | 0 | 7 | 97 | 1 | 0 |
| 69 | 2017-04-28 | 1943-08 | 1 | 1 | 1 | 133 | 39 | 1 | 0 |
| 70 | 2017-04-28 | 1941-06 | 0 | 0 | 0 | 186 | 2 | 1 | 1 |
| 71 | 2017-04-28 | 1945-02 | 1 | 0 | 1 | 104 | 46 | 1 | 0 |
| 72 | 2017-05-01 | 1944-05 | 0 | 0 | 0 | 116 | 48 | 1 | 0 |
| 73 | 2017-05-01 | 1960-04 | 1 | 0 | 1 | 5 | 98 | 1 | 0 |
| 74 | 2017-05-03 | 1934-04 | 1 | 0 | 1 | 108 | 40 | 0 | 0 |
| 75 | 2017-05-05 | 1950-02 | 1 | 0 | 0 | 123 | 42 | 1 | 0 |
| 76 | 2017-06-02 | 1937-05 | 0 | 0 | 0 | 61 | 72 | 1 | 0 |
| 77 | 2017-06-02 | 1945-02 | 0 | 1 | 0 | 110 | 55 | 1 | 1 |
| 78 | 2017-06-19 | 1956-06 | 0 | 0 | 0 | 76 | 51 | 1 | 0 |
| 79 | 2017-06-20 | 1940-05 | 1 | 0 | 1 | 2 | 99 | 1 | 0 |
| 80 | 2017-06-20 | 1939-10 | 0 | 0 | 0 | 170 | 25 | 1 | 0 |
| 81 | 2017-06-28 | 1956-06 | 0 | 0 | 1 | 77 | 60 | 1 | 0 |
| 82 | 2017-06-29 | 1938-03 | 1 | 0 | 1 | 97 | 45 | 1 | 0 |
| 83 | 2017-06-29 | 1948-06 | 0 | 1 | 1 | 161 | 27 | 1 | 1 |
| 84 | 2017-06-29 | 1942-09 | 1 | 0 | 1 | 115 | 23 | 1 | 0 |
| 85 | 2017-07-03 | 1941-12 | 0 | 0 | 1 | 139 | 25 | 1 | 0 |
| 86 | 2017-07-03 | 1947-10 | 0 | 0 | 0 | 111 | 45 | 1 | 0 |
| 87 | 2017-07-03 | 1971-05 | 0 | 0 | 0 | 5 | 97 | 1 | 0 |
| 88 | 2017-07-04 | 1954-11 | 1 | 0 | 0 | 80 | 63 | 1 | 0 |
| 89 | 2017-07-04 | 1938-09 | 0 | 0 | 1 | 191 | 11 | 1 | 0 |
| 90 | 2017-07-04 | 1967-09 | 1 | 0 | 0 | 138 | 35 | 1 | 0 |
| 91 | 2017-07-05 | 1942-08 | 0 | 0 | 0 | 2 | 99 | 1 | 0 |
| 92 | 2017-07-05 | 1946-09 | 0 | 0 | 1 | 102 | 33 | 1 | 0 |
| 93 | 2017-07-05 | 1964-05 | 1 | 0 | 0 | 53 | 72 | 1 | 0 |
| 94 | 2017-07-06 | 1942-10 | 0 | 0 | 0 | 7 | 9 | 1 | 1 |
| 95 | 2017-07-06 | 1951-06 | 1 | 0 | 1 | 154 | 25 | 1 | 0 |
| 96 | 2017-07-07 | 1944-07 | 0 | 0 | 1 | 119 | 37 | 1 | 1 |
| 97 | 2017-07-07 | 1957-11 | 1 | 0 | 1 | 175 | 19 | 1 | 0 |
| 98 | 2017-08-03 | 1940-09 | 1 | 0 | 1 | 90 | 57 | 1 | 0 |
| 99 | 2017-08-07 | 1942-05 | 0 | 0 | 1 | 130 | 39 | 1 | 0 |
| 100 | 2017-08-07 | 1963-11 | 0 | 1 | 0 | 108 | 42 | 1 | 0 |
| 101 | 2017-08-07 | 1963-04 | 0 | 0 | 1 | 83 | 29 | 1 | 0 |
| 102 | 2017-08-10 | 1936-09 | 1 | 0 | 1 | 9 | 96 | 0 | 0 |
| 103 | 2017-08-17 | 1949-03 | 1 | 0 | 1 | 5 | 98 | 1 | 1 |

Data table 2.

| Patient ID-number | rs4244285 | rs12248560 | rs4986893 | rs2046934 | rs6785930 | rs9859552 | rs13059232 | rs2242480 |
| --- | --- | --- | --- | --- | --- | --- | --- | --- |
| 1 | G:G | T:C | G:G | T:T | ? | T:T | T:T | C:C |
| 2 | G:G | C:C | G:G | T:T | ? | T:T | C:C | C:C |
| 3 | G:A | C:C | G:G | T:T | G:A | G:G | T:T | C:C |
| 4 | G:G | T:C | G:G | T:T | A:A | G:G | T:C | C:C |
| 5 | G:G | T:T | G:G | T:T | ? | T:G | T:C | C:C |
| 6 | G:A | T:C | G:G | C:C | G:G | G:G | C:C | C:C |
| 7 | G:G | C:C | G:G | T:T | G:A | G:G | T:C | T:C |
| 8 | G:A | T:C | G:G | T:T | G:A | T:G | T:T | C:C |
| 9 | G:G | C:C | G:G | T:T | G:A | T:G | T:C | C:C |
| 10 | G:G | ? | G:G | ? | ? | ? | ? | ? |
| 11 | G:G | C:C | G:G | T:T | G:A | G:G | C:C | T:C |
| 12 | G:G | C:C | G:G | T:T | G:A | G:G | T:C | C:C |
| 13 | G:G | C:C | G:G | T:T | G:G | T:G | C:C | T:C |
| 14 | G:A | C:C | G:G | C:T | G:G | G:G | C:C | C:C |
| 15 | G:G | C:C | G:G | C:T | G:A | G:G | T:T | C:C |
| 16 | G:A | C:C | G:G | T:T | G:A | G:G | C:C | C:C |
| 17 | G:G | C:C | G:G | T:T | G:A | G:G | C:C | C:C |
| 18 | G:G | C:C | G:G | T:T | G:A | G:G | C:C | T:C |
| 19 | G:G | T:C | G:G | T:T | G:A | G:G | T:T | C:C |
| 20 | G:G | C:C | G:G | T:T | G:A | T:G | T:T | C:C |
| 21 | G:G | C:C | G:G | T:T | A:A | G:G | C:C | T:C |
| 22 | G:A | C:C | G:G | T:T | G:A | T:G | C:C | C:C |
| 23 | G:G | C:C | G:G | G:G | T:T | G:G | T:C | C:C |
| 24 | G:G | C:C | G:G | T:T | G:G | T:G | C:C | C:C |
| 25 | G:G | T:C | G:G | T:T | G:G | T:G | C:C | T:C |
| 26 | G:G | C:C | G:G | T:T | G:G | T:G | C:C | C:C |
| 27 | A:A | C:C | G:G | T:T | G:A | G:G | T:C | T:C |
| 28 | G:G | T:C | G:G | T:T | G:A | G:G | T:T | T:C |
| 29 | G:G | C:C | G:G | T:T | A:A | G;G | C:C | C:C |
| 30 | G:A | C:C | G:G | T:T | G:A | T:G | C:C | C:C |
| 31 | G:G | T:C | G:G | T:T | G:A | G:G | T:C | C:C |
| 32 | G:G | C:C | G:G | T:T | G:G | T:G | C:C | C:C |
| 33 | G:G | C:C | G:G | T:T | G:G | T:G | T:C | C:C |
| 34 | G:G | C:C | G:G | T:T | G:A | T:G | T:C | T:C |
| 35 | DUPE | T:C | G:G | C:T | G:G | G:G | C:C | T:C |
| 36 | G:G | T:C | G:G | T:T | G:A | G:G | C:C | T:C |
| 37 | G:G | T:C | G:G | T:T | G:A | G:G | T:T | C:C |
| 38 | G:G | C:C | G:G | T:T | G:G | T:G | T:T | C:C |
| 39 | G:A | C:C | G:G | T:T | A:A | G:G | T:C | T:C |
| 40 | G:A | C:C | G:G | T:T | G:G | T:T | T:C | C:C |
| 41 | G:G | C:C | G:G | T:T | G:A | G:G | T:C | C:C |
| 42 | G:G | T:T | G:G | T:T | G:G | T:G | C:C | C:C |
| 43 | DUPE | T:T | G:G | C:T | G:G | G:G | T:C | C:C |
| 44 | G:G | C:C | G:G | T:T | G:A | G:G | T:C | C:C |
| 45 | G:G | T:C | G:G | T:T | G:G | T:G | C:C | C:C |
| 46 | G:G | C:C | G:G | T:T | G:G | G:G | C:C | C:C |
| 47 | G:G | C:C | G:G | C:C | G:G | G:G | C:C | T:C |
| 48 | G:A | C:C | G:G | T:T | G:A | G:G | C:C | T:C |
| 49 | G:G | T:C | G:G | T:T | G:A | T:G | C:C | C:C |
| 50 | G:A | T:C | G:G | T:T | G:A | G:G | T:T | C:C |
| 51 | G:A | C:C | G:G | C:C | G:G | G:G | C:C | C:C |
| 52 | G:G | C:C | G:G | T:T | G:G | G:G | C:C | C:C |
| 53 | G:G | C:C | G:G | T:T | G:A | T:G | T:C | C:C |
| 54 | G:A | C:C | G:G | C:T | G:A | G:G | T:T | C:C |
| 55 | G:G | C:C | G:G | C:T | G:G | G:G | T:C | C:C |
| 56 | A:A | C:C | G:G | C:T | G:G | T:G | C:C | C:C |
| 57 | G:G | C:C | G:G | C:T | G:G | T:G | T:C | T:C |
| 58 | G:A | C:C | G:G | T:T | G:A | G:G | T:C | C:C |
| 59 | G:G | C:C | G:G | T:T | G:A | G:G | T:C | C:C |
| 60 | G:G | C:C | G:G | C:T | G:A | G:G | T:C | C:C |
| 61 | G:G | C:C | G:G | T:T | A:A | G:G | C:C | C:C |
| 62 | G:A | C:C | G:G | T:T | G:A | G:G | T:C | C:C |
| 63 | G:A | C:C | G:G | C:T | G:G | G:G | C:C | T:C |
| 64 | G:A | C:C | G:G | T:T | G:A | T:G | T:C | C:C |
| 65 | G:A | C:C | G:G | C:C | G:G | G:G | T:C | T:C |
| 66 | G:G | T:C | G:G | C:T | G:G | G:G | C:C | C:C |
| 67 | G:G | T:T | G:G | C:C | G:G | G:G | C:C | C:C |
| 68 | G:G | T:C | G:G | T:T | G:A | G:G | T:C | C:C |
| 69 | G:G | T:C | G:G | T:T | G:G | G:G | C:C | T:C |
| 70 | G:A | C:C | G:G | C:T | G:G | G:G | T:C | C:C |
| 71 | G:A | C:C | G:G | T:T | G:A | G:G | T:C | C:C |
| 72 | G:G | C:C | G:G | T:T | G:G | G:G | C:C | C:C |
| 73 | G:A | C:C | G:G | C:T | G:A | G:G | C:C | C:C |
| 74 | G:A | C:C | G:G | T:T | A:A | G:G | T:C | T:C |
| 75 | G:G | C:C | G:G | T:T | G:G | T:G | T:T | C:C |
| 76 | G:G | T:T | G:G | T:T | G:A | T:G | C:C | C:C |
| 77 | G:G | T:C | G:G | T:T | G:G | T:G | T:T | C:C |
| 78 | G:G | C:C | G:G | C:T | G:G | G:G | C:C | C:C |
| 79 | G:G | C:C | G:G | C:C | G:G | G:G | T:C | T:C |
| 80 | G:G | C:C | G:G | T:T | G:G | T:G | T:C | C:C |
| 81 | G:G | C:C | G:G | T:T | G:G | T:G | C:C | T:C |
| 82 | G:G | T:C | G:G | T:T | G:A | T:G | T:C | T:C |
| 83 | G:G | C:C | G:G | T:T | G:G | T:G | C:C | C:C |
| 84 | G:G | C:C | G:G | C:T | G:A | G:G | T:C | T:C |
| 85 | G:A | C:C | G:G | T:T | G:A | T:G | C:C | C:C |
| 86 | G:A | T:C | G:G | C:T | G:A | G:G | C:C | T:C |
| 87 | G:G | T:T | G:G | C:T | G:A | G:G | T:T | C:C |
| 88 | G:G | T:C | G:G | T:T | G:A | T:G | T:C | T:C |
| 89 | G:A | C:C | G:G | T:T | G:A | G:G | T:C | C:C |
| 90 | G:G | C:C | G:G | C:T | G:G | G:G | T:T | C:C |
| 91 | G:G | T:C | G:G | T:T | G:A | G:G | T:C | C:C |
| 92 | G:A | C:C | G:G | C:T | G:A | G:G | T:C | C:C |
| 93 | G:G | C:C | G:G | T:T | G:A | T:G | C:C | C:C |
| 94 | G:G | C:C | G:G | C:T | G:G | G:G | C:C | C:C |
| 95 | G:G | C:C | G:G | T:T | G:G | G:G | T:T | C:C |
| 96 | G:G | C:C | G:G | C:T | G:G | T:T | T:C | T:C |
| 97 | G:A | C:C | G:G | T:T | G:G | G:G | C:C | T:C |
| 98 | G:G | C:C | G:G | C:T | G:A | G:G | T:T | C:C |
| 99 | G:A | C:C | G:G | T:T | G:G | G:G | T:C | C:C |
| 100 | G:G | T:C | G:G | C:T | G:G | G:G | C:C | C:C |
| 101 | G:A | C:C | G:G | C:T | G:G | G:G | T:T | C:C |
| 102 | G:G | T:C | G:G | C:T | G:G | G:G | T:C | T:C |
| 103 | G:G | T:C | G:G | T:T | G:A | G:G | C:C | C:C |
