# Supplementary material for: Clopidogrel responder status is uninfluenced by CYP2C19*2 in Danish patients with stroke
Source: PLoS One. 2020 Dec 28;15(12):e0236260. doi: 10.1371/journal.pone.0236260 (PMC7769274; doi:10.1371/journal.pone.0236260)
Supplement: Attachment — Submitted filename: Response to reviewers.docx [file pone.0236260.s002.docx]

**Response to reviewers.**

Thank you for your thorough review.

Please see responses below. We hope you find them satisfactional.

**Reviewer #1:**

1) It is a pharmacogenetic study that evaluates the influence of CYP2C19, CYP3A4, NR1I2 and PR2Y12 polymorphisms in the response to clopidogrel in patients with a history of stroke or transient ischemic attacks.
   As expect, they found a relationship between CYP2C19 polymorphisms and platelet reaction unit (PRU) but no relation with the other genes. Surprisingly, they did not find a relation of CYP2C19 polymorphisms with high-on-treatment platelet reactivity (HTPR) to clopidogrel, a way to measure no-response to this drug. This can be explain by a methodological error in the measurement of PRU because the incidence of HTPR in other studies varies from 8 to 61% (mean 27%) and the prevalence in this study is 0%. How can we be sure that PRU results are reliable? If there are no HTPR patients, it is impossible to find an association with any factor.

Answer: Most previous studies I clopidogrel HTPR have measured PRU-values in the first few days after an ischemic event (cerebral or cardiac). There is evidence that there is an increased activation of platelets in the acute phase after stroke.

We measured PRU values not in the acute phase (at least 14 days after stroke or TIA) which is probably why we have not found any patients with HTPR.

2) Moreover, HTPR was defined as an PRU value >208 and other studies use a cut-off of >185 (see Saiz-Rodríguez et al. Influence of CYP450 Enzymes, CES1, PON1, ABCB1, and P2RY12 Polymorphisms on Clopidogrel Response in Patients Subjected to a Percutaneous Neurointervention. Clin Ther. 2019 Jun;41(6):1199-1212).

Answer: There are different cut-off-values in different studies. We chose 208 because of the results from the Gravitas trial with 5427 subjects. Furthermore, these results made basis for our power analysis. The Cut-off at 208 was also based on the manufacturers guide at the time that we planned the study, wrote the protocol and through the patient-recruitment and blood-sampling period.

3) On the other hand, the influence of concomitant medication, as proton pump inhibitors (PPI), that inhibit CYP2C19 has not been evaluated. More CYP2C19 wild type patients are receiving PPI (34.3%) than CYP2C19*2 carriers (16.1%) and this difference can influence the comparison of the two groups

Answer:It is discussed whether some PPI influence CYP2C19 activity. More patients in the non-carrier group received PPI than in the carrier-group. PPI would push the groups towards higher PRU-values and therefore more would have HTPR. But still we do not have any.

**Reviewer #2:**

4) Abstract: HTPR: is not explained in the abstract

Answer: Abstract changed.

5) Introduction: The aim of the study sholud be mention in the Intruduction section.

Answer: Aim is mentioned in the introduction. Please see blue highlight.

6) Please indicated the reasosns for investigated the named SNPs: CYP2C19*2 (rs4244285), *3 (rs4986893), and *17 (rs12248560), and CYP3A4*1G (rs2242480).
   NR1I2 (rs13059232), P2RY12 (rs2046934 and rs6785930), and P2Y12 (rs9859552)

Answer: Please see green highlight.

7) Method: The authors should explainne why they use PCR_RFLP mathod and competitive allele-specific PCR (KASP) for genotyping of CYP2C19*2 (rs4244285), *3 (rs4986893) SNPs? By KASP they may genotyped also competitive allele-specific PCR (KASP). I do not understand why they used also PCR-RFLP method

Answer: The study was part of af pHd-programme with required laboratory work. Therefore, some test were made with PCR-RFLP.

8) Why the authors did not exclude the cases with undetermined SNPs.? In Results section: Please check and reformulate „ In 1-4 patients the SNP the examined SNP was undetermined.” It is not clear..... Patient number 10 (from Supplemental material has only two SNPs determined.and 6 undetermined. Why he was not excluded from the study ?

Answer: All patients had several SNP examined. If one SNP was undetermined but other SNPs were determined, the patient was not excluded from analysis from the SNPs that were determined.

Rephrased.

9) The authors should discus also another study performe in europeans patients (189 clopidogrel-treated patients with acute coronary syndromes and noncardiogenic ischemic stroke).
   Mărginean A, et al. The Impact of CYP2C19 Loss-of-Function Polymorphisms, Clinical, and Demographic Variables on Platelet Response to Clopidogrel Evaluated Using Impedance Aggregometry. Clin Appl Thromb Hemost. 2017 Apr;23(3):255-265. doi: 10.1177/1076029616629211. Epub 2016 Jul 9

Answer: Added as reference.
