# Supplementary material for: Clopidogrel responder status is uninfluenced by CYP2C19*2 in Danish patients with stroke
Source: PLoS One. 2020 Dec 28;15(12):e0236260. doi: 10.1371/journal.pone.0236260 (PMC7769274; doi:10.1371/journal.pone.0236260)
Supplement: Attachment — Submitted filename: Response to reviewers june 2020.docx [file pone.0236260.s003.docx]

**Response to reviewers.**

Please see responses below. We hope you find them satisfactional.

**Reviewer #1:**

1) The authors did not find any patient with high-on-treatment platelet reactivity (HTPR) to clopidogrel, when the incidence of HTPR in other studies varies from 8 to 61% (mean 27%). How can we be sure that platelet reaction unit (PRU) results are reliable? The authors did not find any patient with high-on-treatment platelet reactivity (HTPR) to clopidogrel, when the incidence of HTPR in other studies varies from 8 to 61% (mean 27%). How can we be sure that platelet reaction unit (PRU) results are reliable? The authors did not find any patient with high-on-treatment platelet reactivity (HTPR) to clopidogrel, when the incidence of HTPR in other studies varies from 8 to 61% (mean 27%). How can we be sure that platelet reaction unit (PRU) results are reliable? The authors did not find any patient with high-on-treatment platelet reactivity (HTPR) to clopidogrel, when the incidence of HTPR in other studies varies from 8 to 61% (mean 27%). How can we be sure that platelet reaction unit (PRU) results are reliable? The authors did not find any patient with high-on-treatment platelet reactivity (HTPR) to clopidogrel, when the incidence of HTPR in other studies varies from 8 to 61% (mean 27%). How can we be sure that platelet reaction unit (PRU) results are reliable?

Answer: We have followed all instructions from the manufacturer regarding blood sampling and analysis. All blood sampling and analysis were done by staff familiar with the procedures.

2) They justify that they measured PRU values not in the acute phase (at least 14 days after stroke or TIA) which is probably why they have not found any patients with HTPR. This should be commented in the discussion and some references should be included to support this argument. They justify that they measured PRU values not in the acute phase (at least 14 days after stroke or TIA) which is probably why they have not found any patients with HTPR. This should be commented in the discussion and some references should be included to support this argument.

Answer: We believe this is already addressed. Please see yellow highlighted section in the discussion.

3) The influence of PPI treatment should be statistically analysed as well as their interaction with CYP2C19 polymorphisms; this information should be included in results and commented in discussion

Answer: The aim of this study is not to investigate whether different PPI´s affect clopidogrelresponse. The study is not sufficiently powered to investigate different types of PPI´s. We are not aware of any study that has found different PPI´s in patients treated with clopidogrel to have an effect on clinical outcome.
